# Analysis of Pig Farmers’ Preference and Adoption Behavior for Food Safety Information Labels in China

**DOI:** 10.3390/foods12061260

**Published:** 2023-03-16

**Authors:** Yingqi Zhong

**Affiliations:** School of Economics, Zhejiang Gongshang University, Hangzhou 310018, China; zhongyingqi2011@163.com

**Keywords:** food labels, willingness to produce, choice experiment, latent class model

## Abstract

Food labels are used to reduce the inefficiencies that arise from information asymmetry. Since food certification and traceability labels are commonly food safety information labels used in China, it is of great importance to study producer preference and adoption behavior towards these food safety information labels. This study constructs a profile of food labels that includes different levels of four safety information attributes, including place of origin, edible agricultural products conformity certificate, traceability code, and organic/green certification. Based on the primary data of pig farmers in Zhejiang Province and using Random Parameters Logit Model and Latent Class Model, this paper analyzed farmers’ willingness to supply pork with food safety information labels and discussed farmers’ adoption behaviors in the production process. Results indicate that among the four information attributes, farmers were more likely to supply pork with the place of origin and organic/green certification. They had a negative willingness to provide pork with a conformity certificate that is certificated by a third party. The preferences for food safety information labels were heterogeneous among farmers. 13.5% of the farmers belonged to the certification-inclined class, and 37.9% of the farmers were traceability preferred. However, the adoption rate by farmers of pork with traceability labels in production was only 7.69%. Therefore, governments and markets should increase incentives for farmers to participate in the traceability system and encourage farmers to issue certificates, and further strengthen the education and training of farmers.

## 1. Introduction

In recent years, food safety has become a major issue of public concern in China. One of the factors contributing to the incidence of food safety issues is information asymmetry [1]. Due to information asymmetry, consumers cannot assess the safety of food before making a purchase [2]. Food labels are one of the instruments to effectively eliminate information asymmetry, reduce the asymmetry of knowledge on food safety, and prevent food safety risks [3,4]. Through information transmission, food labels can convey information about the safety and quality of food and improve consumers’ perceptions of credibility attribute performance evaluations [5]. This will improve consumers’ confidence in the safety of food and alter their purchasing behavior [6].

Food quality and safety certification traceability systems are commonly used to bridge the information gap between producers and consumers and reduce inefficiencies that arise from information asymmetry [7]. Currently, food safety information labels used in China mainly include the place of origin, green/organic certification, traceability code, and edible agricultural products conformity certificate (hereinafter referred to as conformity certificate). The place of origin information is one of the most important attributes that affect consumers’ food choices [8]. Green/organic certificate label was implemented in the 1990s [9], which aims to certify foods that are produced environment-friendly and safe for human consumption [10]. In 2004, China started to use the food traceability system to facilitate meat traceability. By monitoring the food production process through traceability, it can determine the source of food safety problems and recall the food [11]. The conformity certificate was trial implemented in 2016. Using the practice of industrial certificate, a conformity certificate is a commitment made by producers to consumers for the food quality [12].

In recent years, these food safety information labels have been utilized in blends. For instance, some cities in China have implemented “Conformity Certificate + Traceability Code” and “Conformity Certificate + Place of Origin” labels on a trial basis, aiming to create a composite method for transmitting information on food safety [12]. The efficiency of food safety risk communication by food labels is affected by the food providers. As producers’ behavior plays an important role in the supply of food with information labels, it is crucial to analyze their behavior in adopting these labels.

The rest of this paper is organized as follows. Section 2 is a review of relevant literature, followed by the methodology, including choice experiment design, economic modeling and data collection. Section 4 discussed the findings, and Section 5 summarized the conclusions.

## 2. Literature Review

Since food labels may affect consumers’ purchase behaviors, a considerable body of literature has investigated consumer perception and willingness to pay for food with labels in recent years [13,14,15,16]. Consumers’ awareness of food safety information labels with various attributes is quite different, and their willingness to pay (WTP) varies [17]. Xia and Zeng used a meta-analysis to summarize 96 pieces of research on consumers’ WTP for organic food and concluded that the premium WTP by consumers for organic food ranges from 2.3% to 509.2% [18]. The additional payment of Chinese consumers for traceable pork is 15%–30% [19]. Consumers are more concerned with the identification of quality certification or quality standards than origin certification and traceability labels [20,21]. Loureiro and Umberger also confirmed that consumers pay more attention to the certification of the United States Department of Agriculture safety inspection than the county of origin and traceability information [22]. Compared with test/measurement indicators such as traceability and organic certification, consumers prefer cues such as product origin certification [23].

Consumers’ preference for different information attributes depends on the correlation between information labels and food safety [24]. The more comprehensive the information is, the higher the WTP of consumers [25]. However, too many information labels will increase the difficulty for consumers to process information. For example, BPOM in Indonesia reported that consumers were not paying enough attention to the information on food labels which contained complex terminology and number [26]. Therefore, it does not mean that the more information contained in the food safety information labels, the better. Studies have analyzed food safety information labels with different attributes. Take pork, for example, Chen et al. [27] included the origin attribute in the research of the traceability information attribute system and discussed consumers’ WTP for the place of origin label. Taking origin as an attribute of quality assurance ex-ante, Wu et al. [28] studied consumers’ WTP of three different information attributes: traceability, traceability with authentication, and place of origin.

Adding food safety information labels will increase production costs [29]. Because of costs and other factors, farmers’ preference for pork with information labels on different attributes is different. Schulz and Tonsor identified farmers’ preferences for traceability systems from the USA and found the heterogeneity of preferences as well as the welfare effects of mandating traceability among farmers [30]. The aim of producers’ selection of product attributes is to seek the maximum profit, and producers will not provide such product attributes unless they find it profitable to supply or they are required to do so by the government [31]. In order to incentive farmers to comply with higher product safety and quality standards, a small price premium is needed. Ortega et al. found that a majority of farmers in China are willing to comply with the standards when given a modest product premium [7].

Previous studies mainly focused on consumers’ WTP of different information attributes of pork. However, fewer studies have examined the determinants of food safety information label adoption from the producer’s side. Understanding producer preferences towards food safety information attributes is crucial to improve the food safety systems that incentivize producers to supply the desired food. In this context, the purpose of this paper is to examine pig farmers’ willingness to adopt different food safety information labels and discuss farmers’ supply behavior of pork with different information labels in the actual production. Specifically, the study focuses on four food safety information labels of pork: the place of origin, conformity certificate, traceability code, and green/organic certification. And these labels are divided into different levels depending on how comprehensive the information is. Farmers’ willingness to produce pork with different information attributes and levels is assessed using a field survey with choice experiment, and survey results are analyzed using a Random Parameters Logit Model and Latent Class Model. By analyzing the supply of food safety information labels from the producer’s side, this paper aimed to improve the efficiency of food safety risk communication by food labels in the food industry.

## 3. Methodology

### 3.1. Attributes and Levels Design

Information labels record, preserve and communicate the information about pork in the supply chain. The attributes and corresponding levels were set according to the content conveyed by the information labels [32]. Farmers use the place of origin to inform consumers of the villages or countries where pigs are raised. Because the quality of pork may affect by the geographical environment, locally-produced pigs are more popular among consumers. Therefore, the place of origin label was divided into the “local production” label (LOCAL) and the “production in other locations” labels (OTHER). In addition, the “no place of origin” label (NOPO) was included as a base point. In the pork supply chain, food safety mainly occurs in stages of farming, processing, and marketing. Because farmers do not involve in the marketing stage, the attribute of the traceability code was divided into three levels: “no traceability” label (NOINF), “processing traceability” label (PROCESS), including information of pig buyers, and “farming traceability” label (FARM), including information of pig feeding farmers. The conformity certificate is issued by pig farmers who promise not to use prohibited and restricted veterinary drugs and illegal additives. At present, the conformity certificate in the market is mostly in the form of producer self-inspection and third-party inspection. Therefore, the conformity certificates of pork were divided into three levels: the “no conformity certificate” label (NOCC) as a base point, the “certificate passing by self-inspection” label (SELF), and the “certificate passing by a third-party inspection” label (THIRD). For the certification label, the certified food on the market is mainly divided into “green food” certification and “organic food” certification. Therefore, the certification label was also divided into three levels: “no certification” label (NOGO), “green certification” label (GREEN), and “organic certification” label (ORGANIC).

Adding food safety information labels will increase production costs. The cost depends on the structure of the supply chain and the complexity of the information transmitted [33]. For example, traceable information increased the cost of production by 1%–10% [34]. Two main factors contribute to the cost of food safety information labels. One is the additional labor paid for the information recording, sorting, and archiving of information, as well as the expenses associated with recording tools and equipment. The other is the cost of obtaining certification and conducting the inspection, as well as the additional investment to enhance the production environment and update inputs.

Through pre-research, we calculated the average price of pork from December 2018 to January 2019 and took the average price of hind leg meat as the base price of pork, which is 32 CNY/kg (around 4.8 USD/kg). The hind leg meat was used because it is the common pork product in the market and can be accepted by consumers in most areas of China. Furthermore, there is no large price difference among hind leg meat, and the profit will not be affected by the differences among the pork products [35]. According to previous research, consumers paid an additional price ranging from 15% to 40% for green, organic, and traceable pork. The price of pork with four information labels raised between 25% and 50%. As a result, three price levels were established: 32 CNY/kg, 40 CNY/kg (around 6.0 USD/kg), and 48 CNY/kg (around 7.2 USD/kg). Using effect codes to assign values to the attributes and their corresponding levels of food safety information labels and prices. The design of the attributes and definition of the variables are shown in Table 1.

### 3.2. Choice Experiment Design

**Figure 1:**
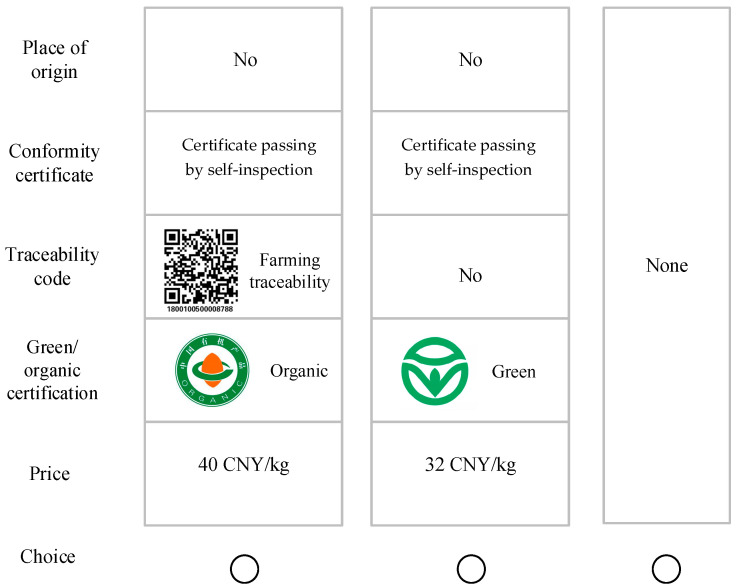
Sample choice set in a task.

Based on the attributes and level setting, 243 (3 × 3 × 3 × 3 × 3) product profiles were constructed, which required respondents to compare and select from 29,403 tasks. Because it is not practically feasible, a fractional factorial design was used. Using Sawtooth Software SSI Web 7.1, 10 versions of the questionnaire were generated at random, and each included 8 tasks. In each task, the respondents were asked to choose one and only one option. The choice sets were displayed to the respondents in color photographs in order to carry out choice experiments in accordance with best practices [22,36]. See Figure 1 for a sample choice set. To control the respondents’ learning effects, the choice sets were presented in a random order [37]. Since omitting the opt-out option would limit respondents’ decision-making and lead them to delay or even refuse to make a choice, “opt-out” was included in the choice set design [38,39]. Therefore, each choice set included two product profiles and an opt-out option.

### 3.3. Economic Modelling

In the choice experiment, farmers selected their preferred alternative from multiple product profiles that consist of different levels of various attributes. Farmers’ willingness to produce pork with different food safety information labels depends on the profit from each information attribute. According to the random utility theory [40], an individual n selects an alternative from a finite set of J alternatives contained in a choice situation k. The probability of an individual n choosing an alternative i is given by:

(1) $$P_{nik}=\mathrm{prob}(V_{nik}-V_{njk}>\varepsilon_{njk}-\varepsilon_{nik};\forall j\neq i)$$

(2) $$V_{nik}=\beta^{\prime }X_{nik}$$

where $V_{nik}$ is a deterministic component which depends on the attributes of an alternative. It is the product of the parameter vector (β) to be estimated and the attribute vector ($X_{nik}$) found in the ith information alternative. The coefficient of the parameter vector to be estimated is also called the part worth of the ith information alternative. $\varepsilon_{nik}$ is a stochastic component, indicating the influence of unobservable factors on producer choice. Assuming that the error $\varepsilon_{nik}$ is independently, identically distributed type Ι, and the producer is homogeneous, then the choice probability would be standard logit, the probability that the producer selects pork with the ith information attribute label given in the Multinomial Logit Model (MNL) is:

(3) $$P_{nik}=\frac{\exp(\beta^{\prime }X_{nik})}{\sum_{j}\exp(\beta^{\prime }X_{njk})}$$

In fact, respondents are heterogeneous in preference for each attribute; therefore, Random Parameters Logit Model (RPL) is appropriate. Since we do not know β and cannot condition on β, the unconditional choice probability is, therefore, the integral of overall possible values of the unknown parameters. The unconditional probability of the sequence of choices is the mixed logit probability formula; thus, the probability of an individual n choosing alternative i in the Random Parameters Logit Model is:

(4) $$P_{nik}=\int \frac{\exp(\beta^{\prime }X_{nik})}{\sum_{j}\exp(\beta^{\prime }X_{njk})}f(\beta )d\beta$$

If f(β) is discrete, the multinomial Logit Model becomes the Latent Class Model (LCM). The heterogeneity of farmers’ preferences can be further analyzed through the Latent Class Model. The probability of an individual n falling into the class t and choosing an alternative i is:

(5) $$P_{nik}=\sum_{t=i}^{T}\frac{\exp({\beta^{\prime }}_{t}X_{nik})}{\sum_{j}\exp({\beta^{\prime }}_{t}X_{njk})}R_{nt}$$

where $\beta^{\prime }$ is the parameter vector of the farmers in class t, $R_{nt}$ is the probability that farmers fall into class t. Assume $Z_{n}$ is the observed value that affects farmers in a certain class t, $\theta^{\prime }$ is the parameter vector of farmers in class t, and r represents the potential class r, therefore:

(6) $$R_{nt}=\frac{\exp({\theta^{\prime }}_{t}Z_{n})}{\sum_{j}\exp({\theta^{\prime }}_{r}Z_{r})}$$

### 3.4. Survey and Data

As the supply of pork with information involved in this study mainly depends on pig farmers and the labels contain information from the breeding stage, this study chose pig farmers as the respondents. The survey was conducted in January 2020 at the main pig breeding districts in Zhejiang Province, where the agricultural product conformity certificate system was on trial in 2016. The districts of the survey were selected with the stratified sampling method, and pig farmers were randomly investigated. The investigation was carried out one-to-one by experienced graduate students with the assistance of staff from the local animal husbandry department. In total, 270 questionnaires were distributed, and 221 questionnaires from the qualified respondents were obtained, with the effective rate of questionnaire recovery being 79.26%. The sample covered 9 districts from 3 cities (that is, Jinhua, Quzhou, and Wenzhou).

The survey obtained more male respondents than females; the percentage of male respondents was 54.30%. The age of respondents was concentrated between 51–70 years old. Compared to the Chinese population, the sample had a low education level; more than 40% of respondents only completed elementary school. The family income of respondents mainly ranged from 30,000 to 60,000 CNY per year. The farming scale of respondents was small, with large-scale farming (that is, with an output of more than 1000 per year) accounting for 23.08%, and the average output size of respondents was 1164. The proportion of specialized farming accounted for 60.63%, and respondents who have been farming for more than 10 years accounted for 71.04%. Descriptive statistics of samples are shown in Table 2.

## 4. Results and Discussion

### 4.1. Farmers’ Preference for Food Safety Information Labels

Using Nlogit 6.0 to estimate farmers’ preference for different levels of food safety information labels with Multinomial Logit Model (MNL)and Random Parameter Logit Model (RPL). The results are presented in Table 3. The third column shows that variances of most variables are significant at the level of 5%, which proves that there is heterogeneity in the preference of farmers. Therefore, the regression results of the random parameter Logit model shall prevail.

Results of both the MNL model and the RPL model show that the variables of OTHER, LOCAL, GREEN, and ORGANIC have significant positive coefficients. Implies that farmers would rather produce pork with labels of the place of origin and organic/green certification than pork without these labels. In addition, farmers’ preference for pork containing processing traceability and certificate passing by self-inspection labels is also significantly higher than that without information labels. These results are consistent with the findings of Ortega et al. [7], which concluded that farmers’ optimal decision in China is adopting environmentally sustainable practices and avoiding using antibiotics. However, farmers’ willingness to produce pork with a certificate passing by a third-party inspection is negative, and they are unwilling to produce pork containing farming traceability labels. The possible reason is that adding certificate labels passing by a third-party inspection and farming traceability label may raise farmers’ production costs; however, consumers’ awareness and acceptance of the pork with these labels are low [8], leading to a low premium making farmers prefer not to provide them.

### 4.2. Preference Heterogeneity of Farmers for Different Food Safety Information Labels

Using Latent Class Model (LCM) to analyze farmers’ preference heterogeneity, the results are shown in Table 4. Farmers could be divided into three classes: those who value green/organic certification labels (certification inclined), those who value traceability labels (traceability preferred), and those who value place of origin labels (origin concerned). 13.5% of the respondents belong to the first class, and the coefficient of green/organic labels is significantly positive, which reveals that farmers in this group obtain profit from certification labels. So that we refer to this class of farmers as “certification inclined.” The second class, which accounts for 37.9% of the respondents, could be referred to as “traceability preferred” farmers, for the coefficient of both effect of processing traceability and farming traceability of this group is significantly positive. The third class, which comprises the vast majority of the respondents (48.6%), is characterized by farmers who put more consideration in place of origin. The coefficient of local production and production in other locations is positive and significant at the 1% level, and they can be referred to as “origin concerned.”

### 4.3. The Order of Importance of Food Safety Information Labels by Farmers

On the basis of the proportion of the difference between the highest and the lowest part-worth of each food safety information attribute and the sum of all the differences, this study calculated the order of importance of the four information attributes based on farmers’ production preference. Table 5 shows the four information attributes in the following order: green/organic certification (56.36%), place of origin (23.72%), traceability code (14.82%), and compliance certificate (5.10%).

It is interesting to note that among these four information attributes, farmers rank green/organic certification label and conformity certificate label as the most important and the least important, respectively. One possible reason is that farmers’ preference for different information labels mainly depends on consumers’ acceptance and the premium they pay for the pork with these labels [41]. The conformity certificate is a food safety information label on the trial, and consumers do not have a clear understanding of this label [42]. Because consumers’ awareness and acceptance are low, farmers believe it to be of the least importance. However, consumers are relatively familiar with the organic/green labels, so farmers are more prefer to produce pork with certification labels [18,43].

### 4.4. Farmers’ Adoption Behavior of Food Safety Information Labels

Farmers’ adoption behavior in practical productions is shown in Table 5. In general, the proportion of farmers who produce pork with these labels accounts for 56.56% of the investigated samples. The majority of them (30.77%) produce pork with conformity certificate labels. And the proportion of farmers who produce pork with the place of origin, traceability, and green/organic certification label accounts for 16.29%, 7.69%, and 1.81%, respectively. By comparing the production intention of farmers, it is found that farmers have the highest preference for producing pork with green/organic certification labels, but only 1.81% of farmers adopted the green/organic certification label in their production behavior. Farmers have the least preference for the conformity certificate label, but the proportion of adoption behavior in practice is the highest.

Farmers’ preference for information labels is inconsistent with their adoption behavior in practice. The production costs, application processes, convenience, and productivity of providing information labels may all have an impact on farmers’ adoption behavior [44]. Farmers prefer to produce pork with green/organic certification labels because these products can bring them higher profits. Green/organic pork, on the other hand, must pass the certification by a specific certification body, and the application and certification procedures are complicated and time-consuming [45]. Farmers are willing to adopt these labels. However, the majority of farmers have not reached the standard that certification requires and have not yet gotten the certification in reality. Resulting in a low adoption rate of green /organic labels in the production practice. In comparison to the green/organic certification label, adding the conformity certificate label is simple [42]. The conformity certificate label is easy to be obtained, and it is issued by pig farmers. Farmers are required to promise not to use prohibited veterinary drugs and illegal additives and to comply with the withdrawal period of veterinary drugs [12]. Therefore, the adoption ratio of the conformity certificate label by farmers is much higher. Similarly, farmers prefer to produce pork with traceability codes, but only 7.69% of the respondents adopt traceability codes during the production practice.

### 4.5. Implications

Understanding farmers’ preference and their adoption behavior for food safety information labels are important because it can help improve regulation policies and rebuild consumer confidence in the food industry. The inconsistency between farmers’ preferences and behavior reduced the efficiency of food safety risk communication by information labels. Therefore, to improve farmers’ willingness to produce pork with certain food safety information labels, the market should improve the incentives for farmers to make up for the increase in production costs. Government should provide subsidies for farmers to increase their motivation to participate in the traceability system and issue conformity certificate labels. Furthermore, to promote farmers’ adoption behavior, governments should simplify the certification and approval procedures of green/organic certification labels, improve the convenience of traceability code information recording and strengthen the education and training of farmers. Farmers should provide food safety information labels with a higher level of information to meet the requirements of the market. Furthermore, they should participate in the education and training of information recording and comply with the requirements for recording and issuing information labels.

## 5. Conclusions

This research analyzed farmers’ preference and their adoption behavior for different food safety information labels. The results show that farmers’ preference for pork with labels of the place of origin, green/organic certificate, processing traceability and certificate passing by self-inspection is significantly higher than that without these information labels. However, their perception does not associate with their adoption behavior. Farmers consider green/organic certification as the most important label, but only 1.81% of the surveyed farmers adopt green/organic certification in the production practice. For the conformity certificate label, although most farmers consider it as the least important, there are more than 30% of the surveyed farmers provide it in the actual production.

This study contains some limitations. The results are based on data obtained from a field survey, and the limitation on sample size and the sample selection may result in biased results. In addition, the results show that farmers’ perception is inconsistent with their adoption behavior, but the study did not analyze the reason for the inconsistency. One possible reason is farmers’ adoption behavior is not only based on the consideration of production cost but also on the convenience of the information recording and the efficiency of corresponding certification and approval procedures. Even in the case of high preference of farmers, they may be reluctant to supply pork with these labels unless the label issuance and approval procedures are convenient. In order to confirm this reason, further investigation and research are needed.

## Figures and Tables

**Table 1:** Attributes design and variables definition of Choice Experiment.

Information Attributes	Attribute Levels	Variables Name	Variables Assignment
Place of origin	No place of origin	NOPO	OTHER = −1, LOCAL = −1
	Production in other locations	OTHER	OTHER = 1, LOCAL = 0
	Local production	LOCAL	OTHER = 0, LOCAL = 1
Traceability code	No traceability	NOINF	PROCESS = −1, FARM = −1
	Processing traceability	PROCESS	PROCESS = 1, FARM = 0
	Farming traceability	FARM	PROCESS = 0, FARM = 1
Conformity certificate	No conformity certificate	NOCC	SELF = −1, THIRD = −1
	Certificate passing by self-inspection	SELF	SELF = 1, THIRD = 0
	Certificate passing by a third-party inspection	THIRD	SELF = 0, THIRD = 1
Green/organic certification	No certification	NOGO	GREEN = −1, ORGANIC = −1
	Green certification	GREEN	GREEN = 1, ORGANIC = 0
	Organic certification	ORGANIC	GREEN = 0, ORGANIC = 1
Price	Base price	PRICE	32 CNY/kg (4.8 USD/kg)
	Middle price, rise 25%	MPRICE	40 CNY/kg (6.0 USD/kg)
	High price, rise 50%	HPRICE	48 CNY/kg (7.2 USD/kg)

**Table 2:** Descriptive statistics of sample characteristics.

Variables	Categories	Number of Respondents	Percentage of Sample (%)
Gender	Male	120	54.30
	Female	101	45.70
Age	Under 30	3	1.36
	30–50	62	28.05
	51–70	136	61.54
	Above 70	20	9.05
Education	Elementary school education and under	89	40.27
	Junior high school	81	36.65
	High school (including secondary occupation education)	39	17.65
	College (including higher vocational technology education)	9	4.07
	Bachelor’s degree and above	3	1.36
Family income	Under 30,000 CNY (4500 USD) per year	57	25.79
	30,000–60,000 CNY (4500–9000 USD) per year	69	31.22
	60,000–100,000 CNY (9000–15,000 USD) per year	45	20.36
	100,000–150,000 CNY (15,000–22,500 USD) per year	32	14.48
	Above 150,000 CNY (22,500 USD) per year	18	8.14
Output of pigs	Under 30	14	6.33
	31–100	72	32.58
	101–1000	84	38.01
	Over 1000	51	23.08
Farming year	Under 10	64	28.96
	11–30	114	51.58
	Over 30	43	19.46
Specialization	Yes	134	60.63
	No	87	39.37

**Table 3:** Famers’ preference for different food safety information labels.

Variables	Multinomial Logit Model		Random Parament Logit Model	
	Coefficients	Standard Error	Coefficients	Standard Error
OTHER	0.171 ***	0.047	0.224 ***	0.060
LOCAL	0.184 ***	0.050	0.244 ***	0.061
SELF	0.165 ***	0.047	0.160 ***	0.057
THIRD	−0.016	0.049	−0.059	0.060
PROCESS	0.230 ***	0.047	0.288 ***	0.056
FARM	−0.004	0.048	0.005	0.060
GREEN	0.397 ***	0.048	0.556 ***	0.080
ORGANIC	0.435 ***	0.049	0.558 ***	0.061
PRICE	0.114 ***	0.017	0.147 ***	0.060
ASC	0.553	0.354	1.231 ***	0.061
NSOTHER	—	—	0.295 ***	0.080
NSLOCAL	—	—	0.295 ***	0.080
NSSELF	—	—	0.104	0.096
NSTHIRD	—	—	0.245 **	0.118
NSPROCESS	—	—	0.188 *	0.127
NSFARM	—	—	0.029	0.125
NSGREEN	—	—	1.018 ***	0.090
NSORGANIC	—	—	0.080	0.129
Log-likelihood	−1360		−1300	
R^2^	0.130		0.311	
AIC	2741		2634	
N	221		221	

Note: ***, **, and * indicate that the parameters are significant at 1%, 5%, and 10% levels, respectively. ASC represents the opt-out option, NSOTHER, NSLOCAL, NSSELF, NSTHIRD, NSPROCESS, NSFARM, NSGREEN, and NSORGANIC represent the variance of the variables OTHER, LOCAL, SELF, THIRD, PROCESS, FARM, GREEN, and ORGANIC respectively.

**Table 4:** Famers’ preference heterogeneity for different food safety information labels.

Variables	Class1 Certification Inclined		Class 2 Traceability Preferred		Class 3 Origin Concerned	
	Coefficients	Standard Error	Coefficients	Standard Error	Coefficients	Standard Error
OTHER	2.555 **	1.060	0.133	0.184	0.355 ***	0.093
LOCAL	−0.056	0.542	0.644 ***	0.182	0.479 ***	0.112
SELF	2.601 ***	0.920	0.235	0.185	0.504 ***	0.100
THIRD	−5.478 ***	1.838	−0.115	0.193	−0.072	0.096
PROCESS	3.113 ***	1.086	0.332 *	0.180	0.475 ***	0.094
FARM	−5.308 ***	1.855	0.534 ***	0.207	−0.183 *	0.104
GREEN	4.948 ***	1.449	3.714 ***	0.386	−0.936 ***	0.153
ORGANIC	2.597 **	1.031	0.202	0.292	1.412 ***	0.148
PRICE	4.267 ***	1.363	−0.024	0.078	0.101 ***	0.034
ASC	4.899	1.522	−0.896	1.574	−0.713	0.708
Class Prob.	0.135		0.379		0.486	

Note: ***, **, and * indicate that the parameters are significant at 1%, 5%, and 10% levels, respectively.

**Table 5:** Comparison of farmers’ production preferences and behaviors for different information labels.

Information Labels	Production Preference (%)	Production Behavior (%)
Place of origin	23.72	16.29
Conformity certificate	5.10	30.77
Traceability code	14.82	7.69
Green/organic certification	56.36	1.81
Total	100	56.56

## Data Availability

Data are contained within the article.
